# Deep learning-assisted detection of lymph node metastases in bladder cancer

**DOI:** 10.1186/s13000-026-01821-8

**Published:** 2026-08-11

**Authors:** Adam Gorczynski, Balazs Tarján, Noora Neittaanmäki

**Affiliations:** 1 Department of Clinical Pathology, Sahlgrenska University Hospital, Gothenburg, Region Västra Götaland Sweden; 2 Path AiD AB, Landvetter, Sweden; 3 Department of Laboratory Medicine, Institute of Biomedicine, Sahlgrenska Academy, University of Gothenburg, Gothenburg, Sweden

**Keywords:** Artificial intelligence, Bladder cancer, Lymph node metastases, Whole slide images, Deep learning

## Abstract

**Background:**

Bladder cancer is the most common malignancy of the urinary tract and is often treated with radical cystectomy with lymph node dissection, followed by a thorough pathological evaluation of the dissected nodes. This is crucial for both accurate prognosis and successful treatment. However, histopathological lymph node examination is labor-intensive and time-consuming.

**Aim:**

To develop a supervised deep learning model for detecting nodal metastases in bladder cancer, and to evaluate its influence on pathologists’ assessment efficiency.

**Methods:**

This study included 100 histopathological slides representing lymph nodes collected between 2015 and 2024 from the Sahlgrenska University Hospital and scanned into whole slide images, divided into training and validation/test sets. The algorithm was trained with 3437 pixelwise annotations. In the validation set, 50 regions containing normal tissue and metastasis were assessed by AI and two specialist pathologists as validators. In the test set, two pathologists reviewed entire slides and measured review times without, and then, after the washout period, with AI assistance.

**Results:**

In the test set, the AI model achieved a sensitivity of 100% and a specificity of 60% for the detection of metastases. For pathologist 1, the median review time decreased from 9 s to 4.2 s with AI assistance, (sign test: Z = -4.73, *p* < 0.001). For pathologist 2, the median review time decreased from 12 to 4 s (sign test: Z = -5.00, *p* < 0.001).

**Conclusions:**

This study demonstrates that an AI model trained on a limited dataset can effectively assist pathologists in the detection of lymph node metastases in bladder cancer while significantly reducing review time. These findings suggest that institutions with limited case volumes may be able to develop and implement locally trained AI models to support routine histopathological assessment.

**Supplementary Information:**

The online version contains supplementary material available at 10.1186/s13000-026-01821-8.

## Background

Bladder cancer (BC) ranks as the ninth most common cancer type globally, with a higher incidence among men and in Western regions. In 2022, there were about 614,000 new cases and 220,000 deaths from BC which makes it rank 13th in terms of mortality among all deaths from all cancer types [1]. A variety of histological types and subtypes of benign and malignant bladder tumors exist. However, the overwhelming majority belongs to the urothelial cancer category, which can present several morphological subtypes, as well as differentiations towards squamous and glandular lineages, while purely non-urothelial bladder carcinomas, such as squamous cell carcinoma and adenocarcinoma, are considerably less common [2].

Radical cystectomy, in combination with neoadjuvant therapy, is the standard treatment for muscle-invasive bladder cancer (MIBC). As a part of the surgical procedure, regional lymph node (LN) dissection is routinely performed. LN metastases at the time of radical cystectomy are associated with markedly poorer outcomes, with historic series showing substantially lower short- and long-term survival in node-positive patients compared with node-negative patients [3–4]. However, LN dissection performed during radical cystectomy improves survival outcomes in patients regardless of pN stage [5]. Given its central prognostic and therapeutic relevance, accurate evaluation of LNs for metastatic involvement is essential but represents a labor-intensive process, contributing substantially to diagnostic workload and turnaround time [6–8].

The adoption of whole slide imaging (WSI) allows for application of advanced digital tools in pathological diagnostics, including artificial intelligence (AI). AI-based approaches have demonstrated strong potential in computational pathology, including the detection of primary tumors, classification of histological subtypes, and prediction of molecular alterations across various cancer types [9–11]. Prior studies have shown that automated image analysis systems can identify LN micrometastases previously missed by pathologists on immunohistochemistry-stained slides [12]. Deep learning (DL) algorithms have also achieved diagnostic performance comparable to that of pathologists in detecting LN metastases in breast cancer, with some models outperforming multiple pathologists in conditions mimicking the time-constraints of the routine diagnostics [13]. Additionally, AI-based methods have demonstrated high performance in detecting LN metastases in lung cancer, including rare variants [14].

AI has also been explored for various applications in bladder cancer pathology. An AI-based model was developed to distinguish non-MIBC from MIBC and achieved high accuracy and specificity, being outperformed only by senior pathologists [15]. Moreover, assessment of the WSIs was faster with the model than by unassisted pathologists. In another study DL models showed the ability to identify molecular subtypes of BC [16]. A recent multicenter study has demonstrated the potential of DL for detecting LN metastases in bladder cancer based on 8,177 WSIs derived from more than 20,000 LNs [17]. While such data-heavy studies establish the technical potential of AI, their applicability to routine pathology practice of smaller institutions may be limited by restricted digital infrastructure, case volumes, and annotation resources. This raises the question of whether LN assessment efficiency can be improved by developing an AI model intended specifically for in-house use within a single institution.

Accordingly, the aim of this study is to develop a supervised DL model using a limited number of WSIs and to evaluate whether AI assistance can accelerate LN assessment in bladder cancer without compromising diagnostic sensitivity.

## Methods

### Material

Patients with BC who underwent radical cystectomy with LN dissection between 2015 and 2024 at Sahlgrenska University Hospital were eligible for inclusion, provided that a representative hematoxylin-eosin (HE) stained glass slide from formalin-fixed paraffin embedded tissue was available for scanning. All patients with LN metastases, stage pN1-3 and a random selection of patients without LN metastases (pN0) who underwent the surgery was included.

Patients were excluded if the primary tumor was not urothelial carcinoma, squamous cell carcinoma, or adenocarcinoma, or if the morphology of the metastatic LN was inconsistent with that of the primary bladder tumor. The ground truth was derived from the original pathology reports, with all slides re-evaluated by an experienced uropathologist (AG). A single slide per case was included in the study, randomly selected from all glass slides containing LN metastases for a given case, or from all HE slides containing LNs in cases negative for metastatic disease.

In total, 102 HE slides, 71 with nodal metastases and 31 without, were collected, anonymized and subsequently scanned digitally using whole-slide imaging in 40x mode (0.23 μm/pixel, 20x objective lens) using the Nanozoomer S210 Digital Slide Scanner (Hamamatsu Photonics K.K., Shizuoka, Japan). Two slides needed to be excluded due to poor quality. The remaining WSIs were uploaded to the Aiforia Create platform (version 6.9; Aiforia Technologies, Helsinki, Finland), where a DL model was developed de novo without prior pretraining.

### Training

The training set included 66% of the total slides with 48 WSI with LN metastases and 18 slides of LNs without metastases. The training annotations were performed exclusively on the training set while validation and test WSIs remained unannotated during model development. The annotations were provided as pixel-level segments by an expert pathologist.

**Fig. 1:**
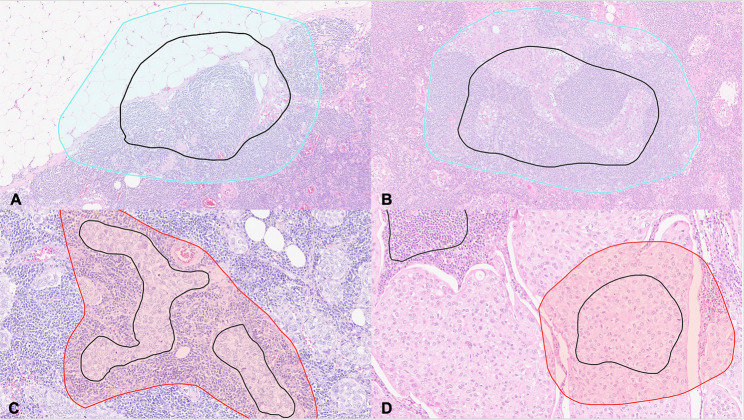
Examples of annotations used for AI training. The tissue layer was used to distinguish tissue from non-tissue (e.g., debris or artifacts), while the metastasis layer trained the AI to differentiate normal tissue from tumor. **A**, **B** Tissue annotations labeled in blue within the tissue layer. **C** Metastasis annotation labeled in red within the metastasis layer. **D** Metastasis annotation labeled in red, with an additional normal tissue annotation in the upper left corner

The deep learning model was based on convolutional neural networks (CNNs) and produced pixel-wise semantic segmentation, classifying each pixel as either foreground or background. Two separate layers were created on the AI platform: a tissue layer and a metastases layer (Fig. 1). The tissue layer was used to distinguish tissue from non-tissue elements such as debris or artifacts, whereas the metastasis layer was used to differentiate metastatic tumor tissue from normal tissue within LNs.

During the training, a total of 3437 training regions (pixel-level annotations) were manually annotated. A total of nine training sessions were conducted prior to the finalization of the AI model, with each training session providing an initial AI performance result. Additional annotations were added after each session to improve the model.

### Validation

The validation was performed on a separate, unannotated validation set prior to testing. A total of 50 manually drawn validation regions were used, which included 16 regions with metastasis, 32 regions with normal tissue and 2 regions containing both. Normal tissue regions included a variety of tissue types found in normal lymph nodes. The validation was assessed by comparing the AI analyses with an independent evaluation performed by two pathologists.

### Testing

The test set included 29 unannotated WSIs (19 slides with LN metastases and 10 without). Two pathologists with over 10 years of diagnostic experience independently reviewed all WSIs in two rounds. In the first round, slides were reviewed without AI assistance, and assessment time was self-recorded for each WSI using a stopwatch. After a two-week washout period, the same slides were reassessed with the AI assistance, and the review times were recorded again. Assessment time was defined as the interval from opening the WSI to establishing a final diagnosis (metastatic or non-metastatic). During the second round, the model-generated mask was displayed at the time of WSI opening.

The pathologists were blinded to the original diagnosis and each other’s evaluation during both rounds. The order of the WSIs was randomized prior to each round to minimize order bias. The model performance was additionally evaluated independently against the ground truth.

### Statistical analyses

The data was analyzed using IBM SPSS Statistics for Windows, Version 29.0.2.0 (IBM Corp., Armonk, N.Y., USA) and R version 4.5.2 (R Foundation for Statistical Computing, Vienna, Austria. https://www.R-project.org). Sensitivity, specificity, positive predictive value/precision (PPV), negative predictive value (NPV), accuracy, and F1 score were calculated. 95% confidence intervals for sensitivity, specificity, PPV and NPV were estimated using the Clopper–Pearson exact method. Interrater agreement was assessed using Cohen’s kappa (κ) and Krippendorff’s alpha (α).

Median, interquartile range (IQR) and mean were used to describe review time results. The Shapiro-Wilk test was used to assess normality of the paired time differences. As distribution was skewed, nonparametric tests (the sign test and the Wilcoxon signed-rank test) were applied for each pathologist separately.

## Results

### Validation

**Fig. 2:**
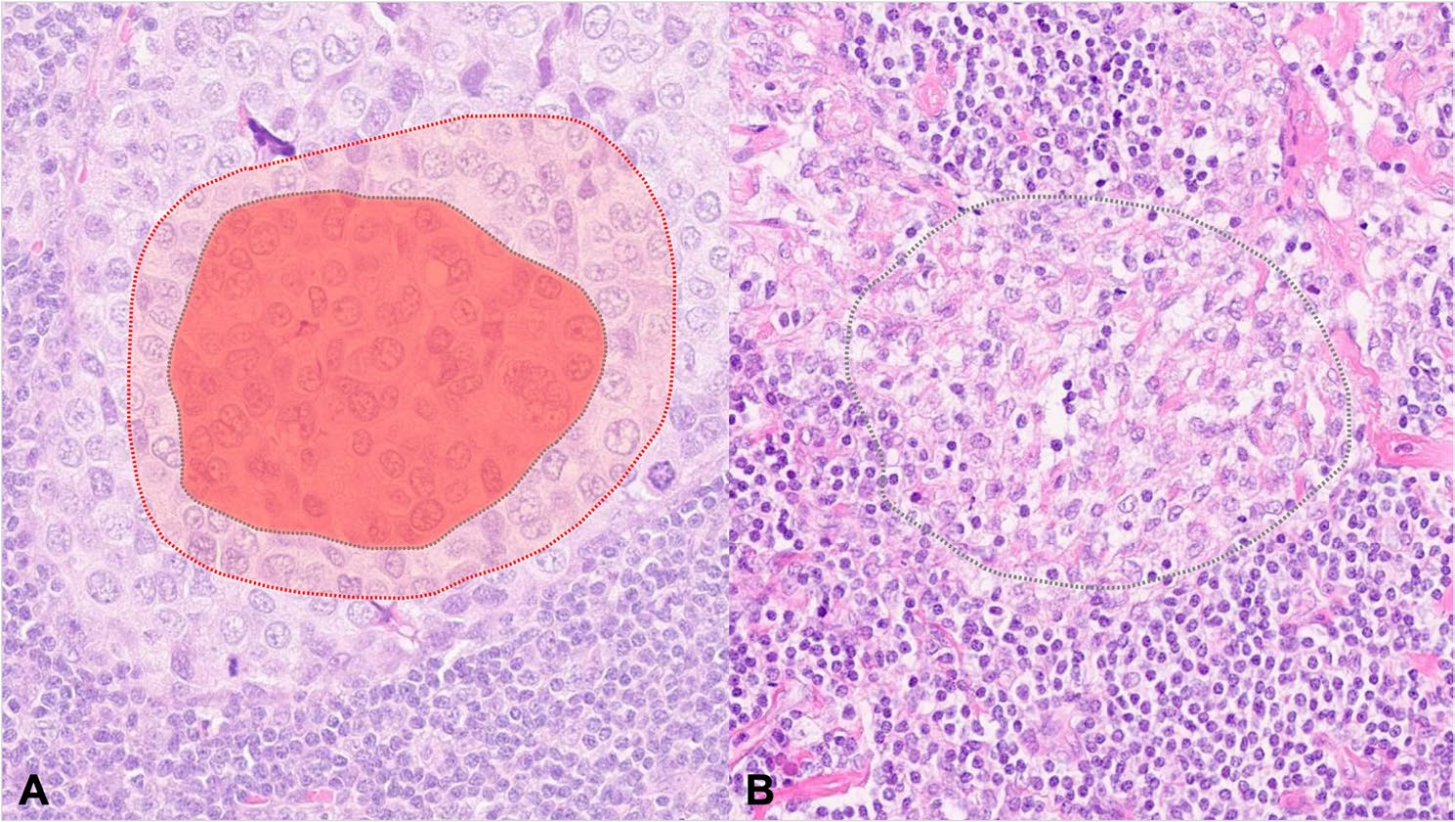
Validation regions used for AI validation. Grey dotted areas indicate validation regions; the AI’s performance was compared to two independent pathologists. **A** Red dotted region marked by the validator, with the red solid area correctly identified by the AI as metastasis. **B** Grey dotted validation region correctly decided as normal tissue by both AI and validator

Validation results are illustrated in Fig. 2. Compared with validator 1, the AI achieved sensitivity of 99.85%, precision (PPV) of 99.78% and F1 score of 99.81%. Corresponding values for validator 2 were 99.85%, 99.49%, and 99.67% respectively. The AI correctly identified all validation regions containing metastases as determined by the validators, and produced no false-positive predictions in regions containing non-metastatic tissue.

### Test results

**Fig. 3:**
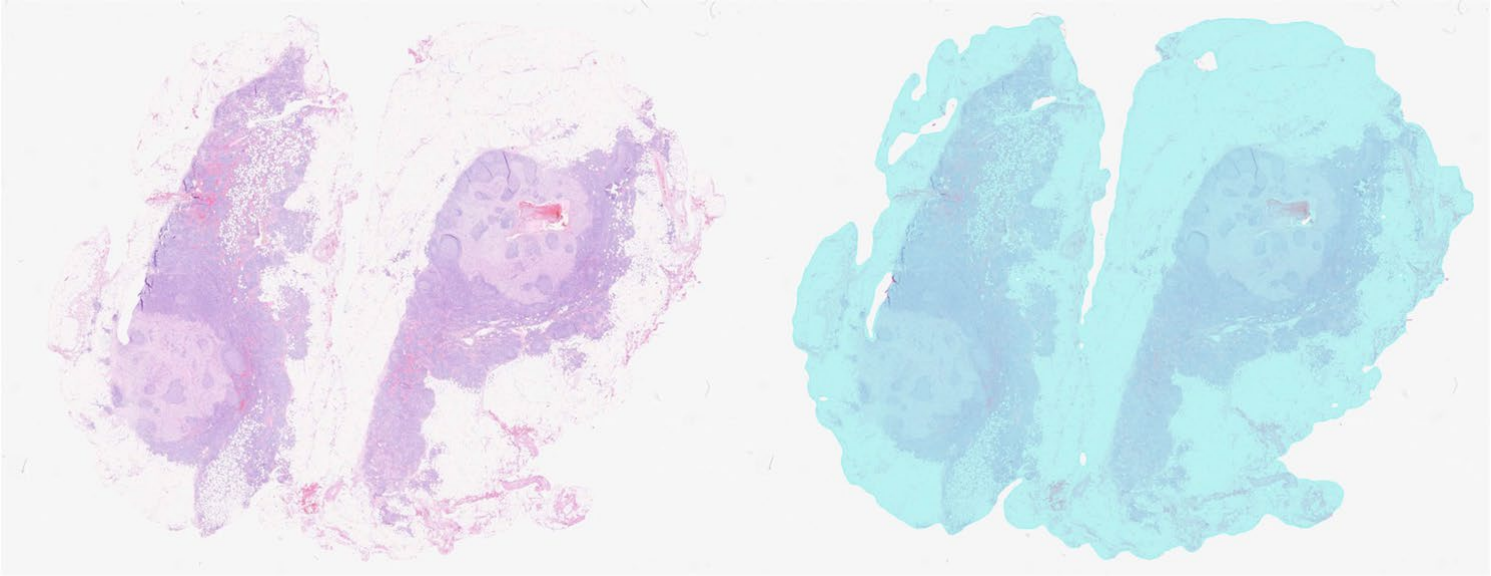
AI detection of tissue. Example of the AI correctly identifying tissue within WSI (blue)

**Fig. 4:**
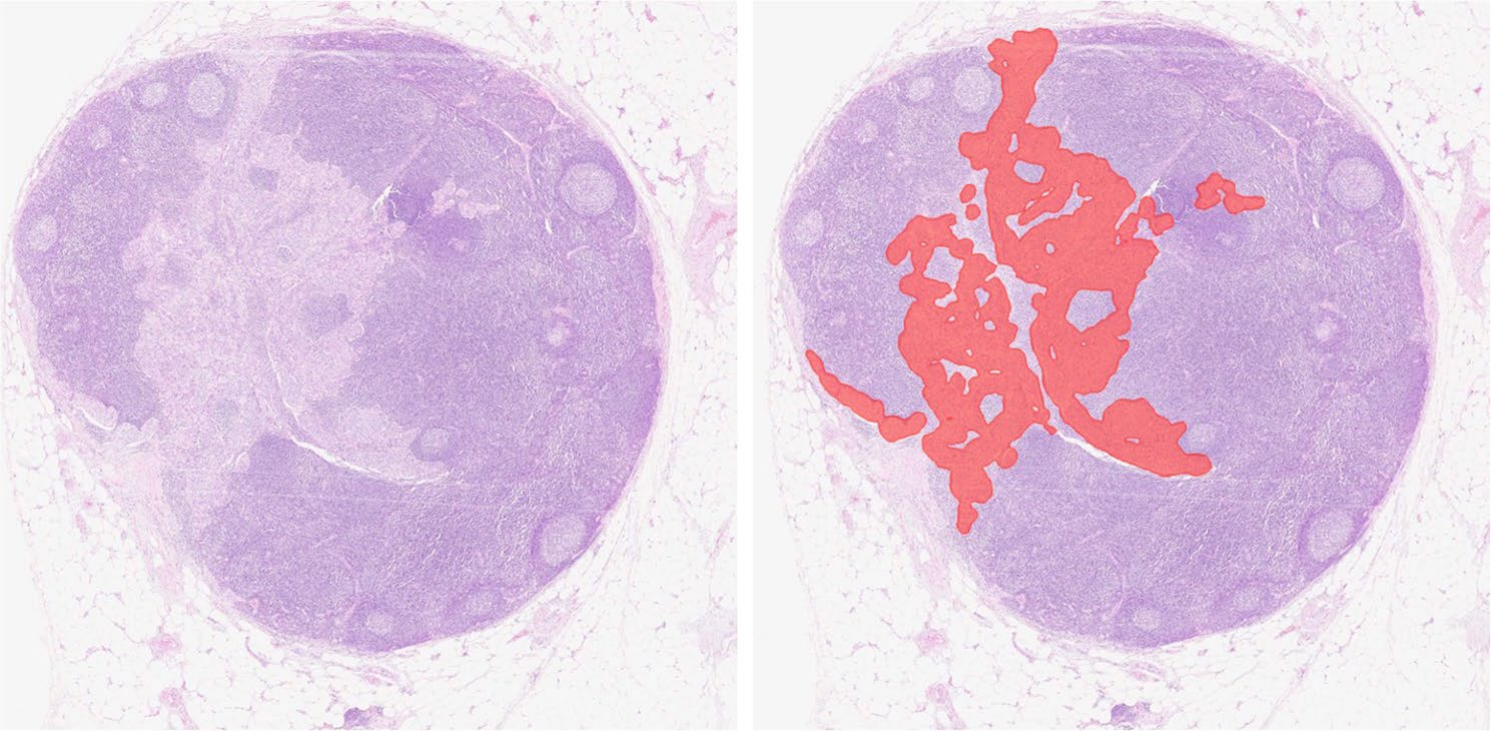
AI detection of metastases. Example of the AI correctly identifying metastatic regions within a lymph node (red)

**Fig. 5:**
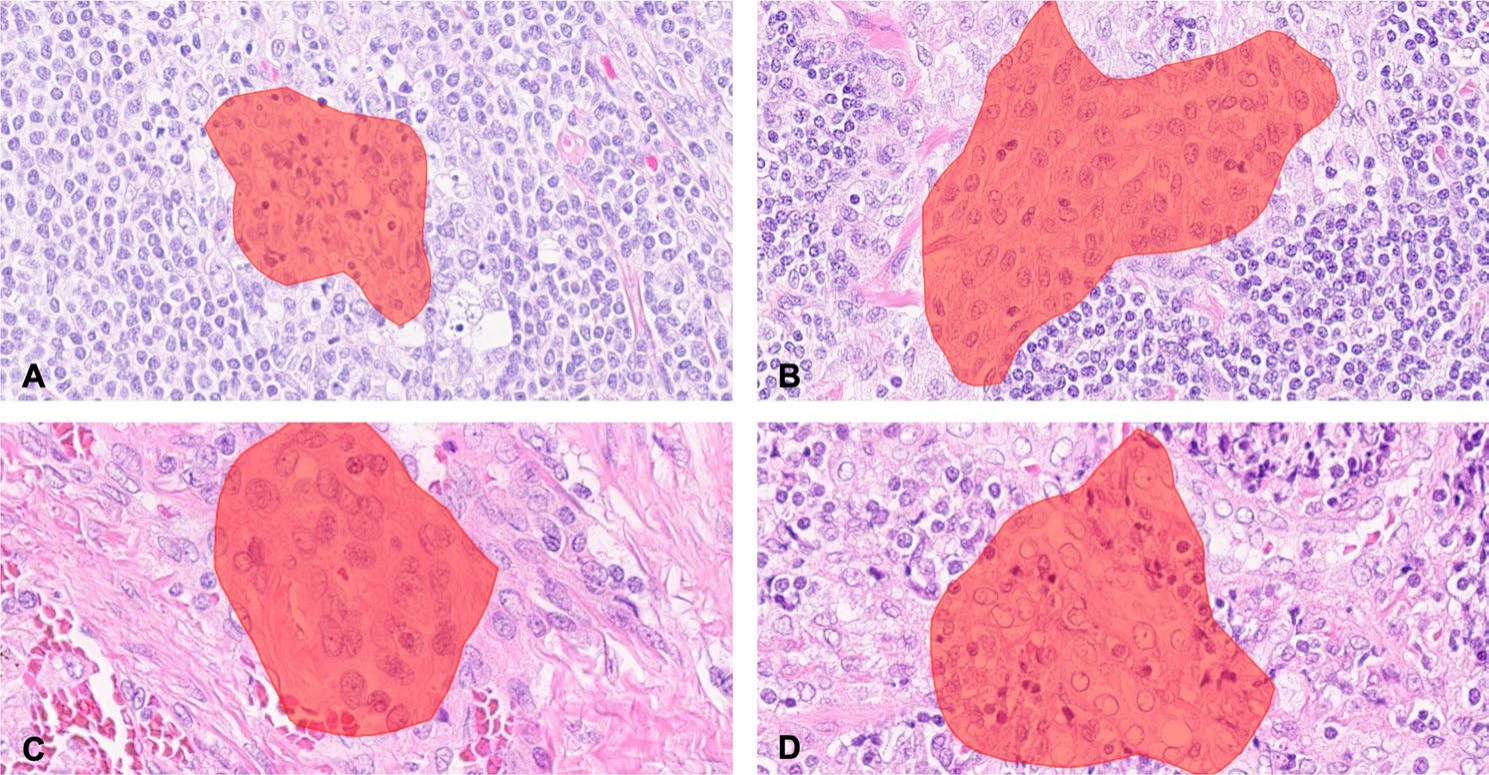
False positive regions labeled by the AI. **A** Germinal center. **B**,**C** Histiocytes. **D** Endothelial cells

The performance of the tissue layer detection is illustrated in Fig. 3. The AI model correctly identified all 19 WSIs containing LN metastases (Fig. 4). Of the 10 negative WSIs, 6 were correctly classified as negative, while 4 contained small false-positive regions labeled as metastatic (Fig. 5). In these cases, the percentage of false metastatic area of total tissue area ranged from 0.0025% to 0.026% (mean: 0.0121%), with 1 to 7 false positive foci per slide.

The interobserver agreement between the two pathologists was perfect (κ = 1.00), with both achieving 100% sensitivity and 100% specificity relative to the ground truth. Agreement between the pathologists and the AI was substantial (α = 0.78, 95% confidence interval (Cl), 0.58–0.95). The AI model demonstrated a sensitivity of 100% (CI 82.4–100%), specificity of 60% (CI 26.2–87.8%), PPV (precision) of 82.6% (CI 61.2–95%), NPV of 100% (CI 54.1–100.0%), accuracy of 86.2%, and F1 score of 90.5%.

**Fig. 6:**
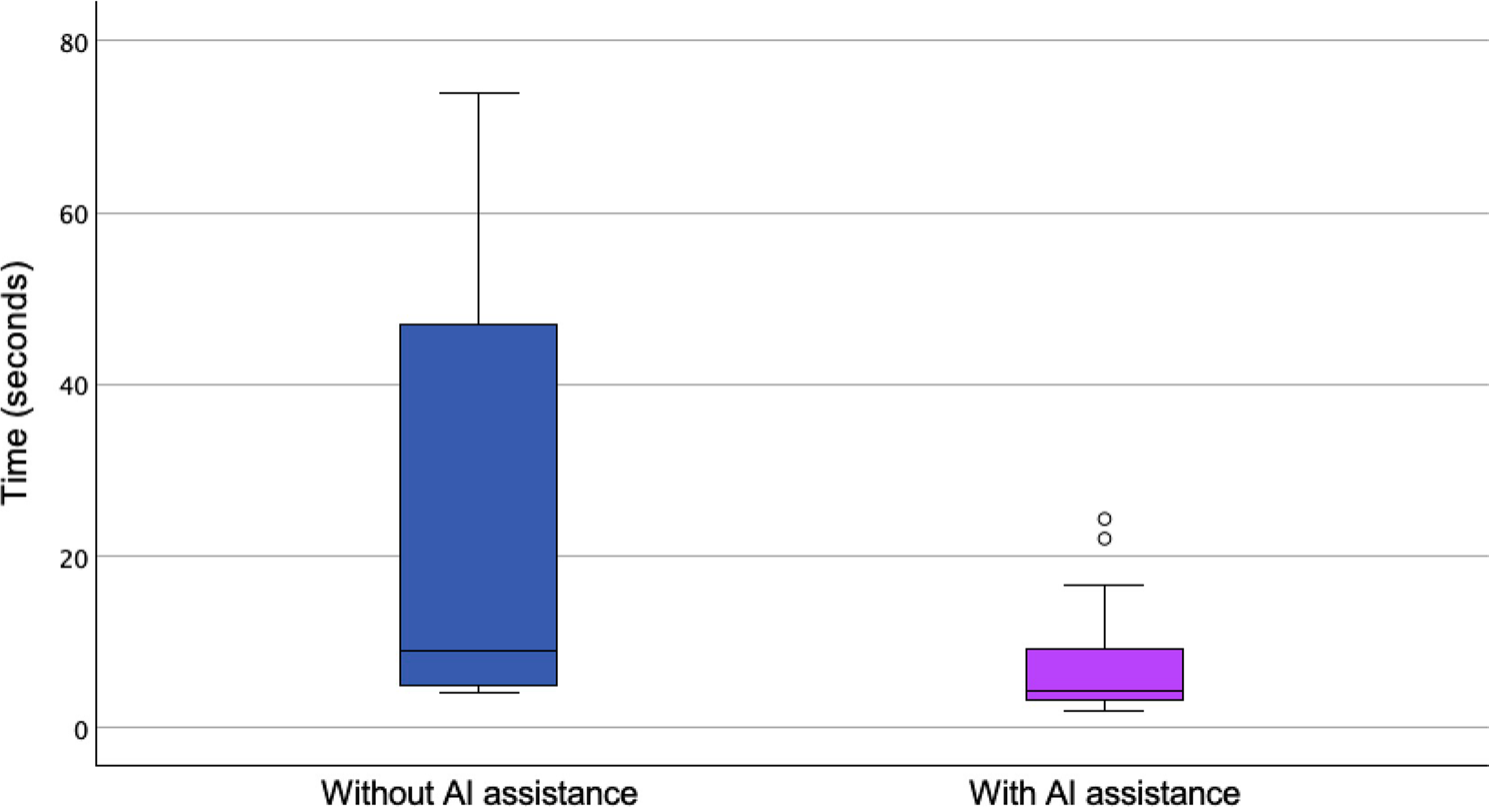
Review times for pathologist 1 with and without AI assistance. Median review time decreased from 9 seconds without AI (left) to 4.2 seconds with AI (right). Individual points beyond the boxplot represent outliers

For pathologist 1, median review time decreased from 9 s (IQR: 5–47.5) without AI to 4.2 s (IQR: 3.25–9.75) with AI (Fig. 6). The median paired difference differed significantly (sign test: Z = -4.73, p < 0.001), and review time was significantly shorter with AI assistance (Wilcoxon signed rank test: Z = -4.44, p < 0.001). Mean review time was reduced by 70.5%, from 24 s to 7 s. For non-metastatic images (n = 10), mean review time decreased by 73.6% (from 49 s to 13 s), and for metastatic images (n = 19) by 62.8% (from 10.7 s to 4 s) (Additional file 1–2). For the non-metastatic cases with false positive areas (n = 4) the mean review time was reduced by 61,2% (from 51 s to 19.8 s).

**Fig. 7:**
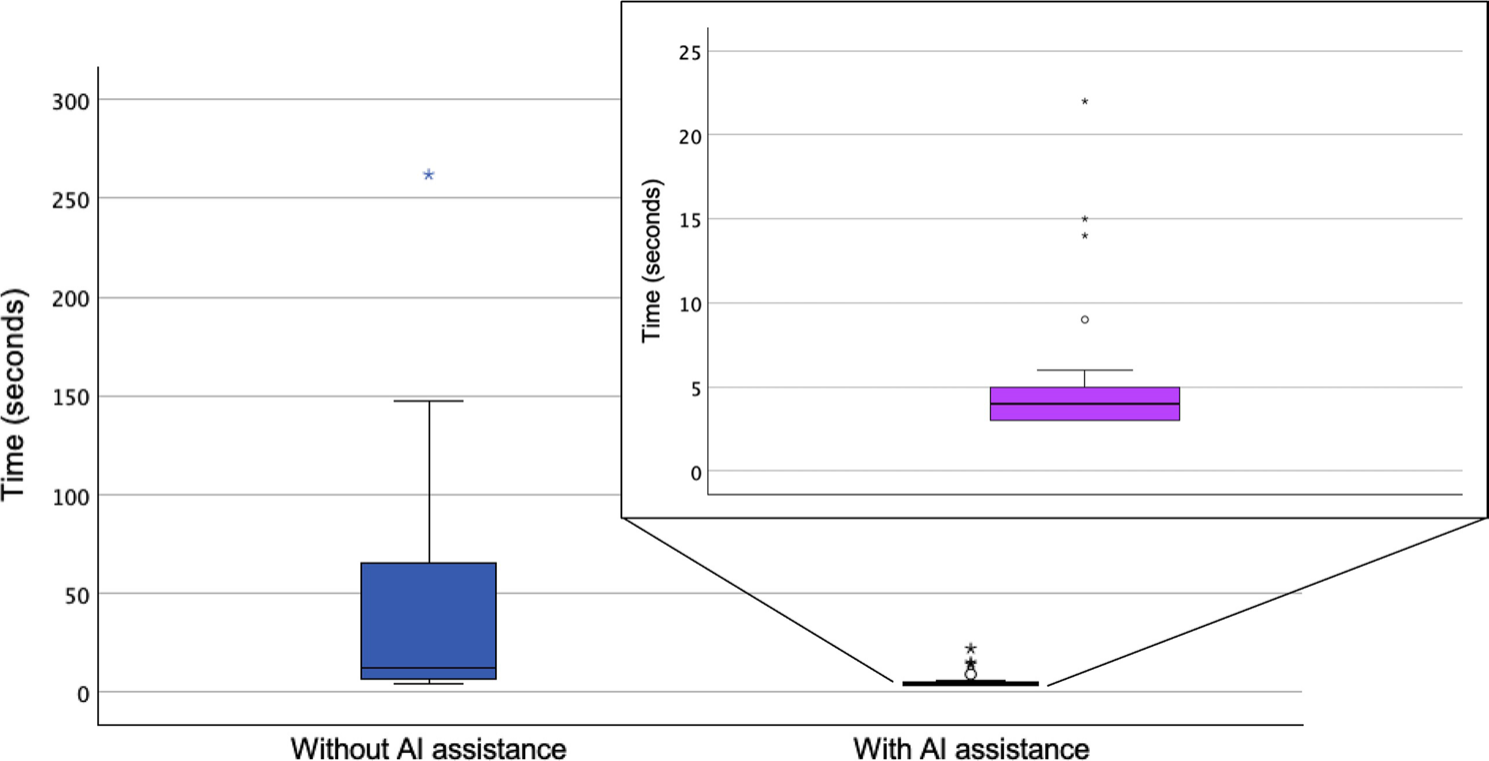
Review times for pathologist 2 with and without AI assistance. Median review time decreased from 12 seconds without AI (left) to 4 seconds with AI (right). Individual points beyond the boxplot represent outliers

For pathologist 2, median review time decreased from 12 seconds (IQR: 7–72) without AI to 4 seconds (IQR: 3–5) with AI assistance (Fig. 7). The median paired difference was significant (sign test: Z = -5.00, p < 0.001; Wilcoxon signed rank test: Z = -4.55, p < 0.001). The mean review time was reduced by 87.5%, from 43 seconds to 5.4 seconds. For non-metastatic images (n=10), mean review time decreased by 92% (from 108 seconds to 8.7 seconds), and for metastatic images (n=19) by 58.6% (from 8.9 seconds to 3.7 seconds) (Appendix Figure 2). For the non-metastatic cases with false positive areas (n=4) the mean review time was reduced by 89,2% (from 138,8 seconds to 15 seconds). 

## Discussion

This study evaluates an AI model for detecting LN metastasis in bladder cancer, focusing on both the algorithm’s performance and its effect on time efficiency. Our findings demonstrate that an AI model trained on a limited, institution-level dataset can accurately assist in LN metastases detection on HE WSIs while significantly reducing review time.

Despite the substantially smaller number of cases and WSIs, our results are consistent with a previously published large multicenter study, which reported a reduction of mean review time by around 23%, while preserving a high diagnostic sensitivity [17]. Similar improvements have also been described for models detecting LN metastases in prostate and breast cancer [18–20].

In the current study, the review times were reduced for both pathologists when aided by AI, with larger reductions observed for non-metastatic slides. Similarly, in breast cancer LN metastases time reductions were observed for negative cases and LNs with micrometastases, but not for macrometastases [19]. The reduction in mean review time may appear modest when considering a single slide, however at a case level, where multiple LNs are examined, this may translate into clinically meaningful time saving. Considering a much higher ratio of negative to positive LNs in routine practice compared to that in the test set, the overall time savings for AI assisted diagnostics may be even more significant in real-world settings than those reported in this study. 

The independently evaluated AI model showed 100% sensitivity and NPV but lower specificity and PPV at the WSI-level, driven by the presence of small false-positive regions, corresponding to blood vessels, germinal centers, histiocytes, macrophages and high endothelial venules (Fig. 5), consistent with model limitations noted in prior studies [17–19]. A similar tendency toward high sensitivity or NPV but lower specificity or PPV has also been previously observed [17–18, 21]. These results, in the context of high accuracy and F1 score, suggest that such AI models may be well suited as screening or triage tools, flagging potentially positive cases for more thorough evaluation by a pathologist. However, these diagnostic performance estimates are based on a limited test set and should therefore be interpreted as preliminary until validated in larger and more diverse cohorts. We also observed a significant reduction in mean review time even in the false positive cases, supporting the model’s usability despite its lower WSI-level specificity. This can be attributed to the high pixel-level specificity, with a maximum of 0.0121% of the total tissue area incorrectly classified as metastatic. Furthermore, we have noticed that in the false positive cases the average round 1 review time was higher than for all non-metastatic cases, which suggests that these WSIs were more challenging even for unassisted pathologists.

One potential limitation of the study is that neither the training nor the test dataset included WSI with only isolated micrometastases. However, given that the smallest discrete metastatic area detected by the model was well below 0,01 mm^2^ (see Additional Fig. 6), the model is likely capable of detecting even smaller metastases.

This model was trained using supervised pixel-level annotations, a method that is considered time-consuming and expensive. While weakly supervised approaches enable training on much larger datasets and have demonstrated strong performance across different cancer types [22], the present study was intentionally designed to reflect the capabilities and limitations of smaller pathology departments. Although a larger dataset can potentially capture greater morphological variability, model performance depends not only on number of cases, but also the quality and diversity of annotations.

Importantly, this study was not intended to develop a model for generalized deployment. No external validation was performed, and the model was designed for in-house use within a single institution. Consequently, performance may be reduced when applied to WSI generated using different scanners or laboratory protocols, as models trained on one scanner may not generalize well to others without adaptation [23–25]. However, our results demonstrate that meaningful efficiency gains can be achieved using locally developed models trained on routine diagnostic material.

An important aspect that may be addressed in future studies is the total resource investment required for model development and deployment, including data preparation, annotation, and system implementation. While our analysis focuses on pathologist review time, future work could evaluate the break-even point at which the cumulative reduction in lymph node WSI review time offsets the initial effort required for model development and implementation.

### Conclusions and implications

This study suggests that an AI model trained on a limited, institution-specific dataset may effectively assist pathologists in lymph node assessment for bladder cancer, maintaining diagnostic sensitivity while potentially improving overall workflow efficiency. These findings indicate that locally developed AI tools may be feasible as supportive systems in routine pathology.

## Supplementary Information

Additional file 1: Review times for images without metastases for pathologist 1. Review times for images without metastases (n=10). Median time of 49 seconds without and 11 seconds with AI assistance.

Additional file 2: Review times for images with metastases for pathologist 1. Review times for images with metastases for pathologist 1 (n=19). Median time of 6 seconds without and 3.7 seconds with AI assistance. Individual points beyond the boxplot represent outliers.

Additional file 3: Review times for images without metastases for pathologist 2. Review times for images without metastases (n=10). Median time of 88 seconds without and 6 seconds with AI assistance.

Additional file 4: Review times for images with metastases for pathologist 2. Review times for images with metastases (n=19). Median time of 7 seconds without and 3 seconds with AI assistance. Individual points beyond the boxplot represent outliers.

Additional file 5: Detailed technical data sheet of the model.

Additional file 6: Examples of isolated tumor areas detected by the model. The model despite not being trained on micrometastases is able to correctly detect small (<0.01 mm2) isolated tumor areas.

## Data Availability

The datasets used and/or analyzed during the current study are available from the corresponding author on reasonable request. The detailed technical data sheet of the model is presented as the Additional File 5.

## Abbreviations

AI: Artificial intelligence
BC: Bladder cancer
CI: Confidence interval
DL: Deep learning
HE: Hematoxylin-eosin
LN: Lymph node
MIBC: Muscle-invasive bladder cancer
NPV: Negative predictive value
PPV: Positive predictive value
WSI: Whole slide image
