# Supplementary material for: Deep learning-assisted detection of lymph node metastases in bladder cancer
Source: Diagn Pathol. 2026 Aug 11;21:58. doi: 10.1186/s13000-026-01821-8 (PMC13459389; doi:10.1186/s13000-026-01821-8)
Supplement: Supplementary file 1 [file 13000_2026_1821_MOESM5_ESM.pdf]

LGL bladder cancer metastases_10000_v9 (1)

Model Origin ID
e3d7a505-498b-886c-8775-0199e881a31e

Model Revision ID
20251203.120354493.841557

Model name
LGL bladder cancer metastases_10000_v9 (1)

Publisher of AI model
8d7d2824-9a8f-4241-8659-6cdca9931484

Timestamp of publication
2025-12-03 12:03:54 (UTC+0)

Project
LGL bladder cancer metastases
ddbdcc77-54d7-88be-86b1-019764076349

Subscription
Sahlgrenska_Neittaanmaki_29042020
6fa30c04-159f-4afd-96d7-19f0ee1991d3

Aiforia software version
Not available

AI engine version
v2

Layer tree

Tissue

Metastases

Layers, Classes and Hierarchy

Layer Name	Classes
Tissue	Tissue
Metastases	Metastases

Parameters for layer: Tissue

**Parameter**	**Value**
Complexity	Very Complex
Layer order	0
Is instance segmentation network	False
Heatmap starting zoom level	200
Heatmap decimation factor	4
Level of detail	50
Gain factor: Tissue	1
Layer type	Region
Allow reporting holes	True
Net structure version	1
Region merging starting zoom level	20
Calculate channel intensity statistics	False
Prevent instance overlap	False
Merge regions across diamond model branches	False
Filtering ancestor layer	0
Desired subpixel resolution	
Consider complete pixels	True
Find parent result area	False
Field of view	300
Weight decay	0.0001
Momentum 1	0.9
Momentum 2	0.999
Mini-batch size	32
Mini-batches per iteration	20
Iterations without progress threshold	100

Parameter	Value
Initial learning rate	1
Maximum learning rate in the end of a training run	0.0001
Learning rate shrink factor	0.5
Scale (minimum)	-5
Scale (maximum)	5
Aspect ratio	5
Maximum shear	5
Luminance (minimum)	-5
Luminance (maximum)	5
Contrast (minimum)	-5
Contrast (maximum)	5
Noise	0
JPG compression percentage	0.5
JPG compression quality (minimum)	40
Blur percentage	0.5
Blur maximum pixels	1
Rotation angle (maximum)	180
Rotation angle (minimum)	-180
Flipping	True
A priori weight	0.5
Desired training window size	4
Minimum training window size pixels	32
Maximum training window size pixels	2048
Desired net structure version	Default
Width scaler	1
Minimum width	2

Parameter	Value
Maximum width	4096

Parameters for layer: Metastases

Parameter	Value
Complexity	Extra Complex
Layer order	1
Is instance segmentation network	False
Heatmap starting zoom level	200
Heatmap decimation factor	4
Level of detail	50
Gain factor: Metastases	1
Layer type	Region
Allow reporting holes	True
Net structure version	1
Region merging starting zoom level	20
Calculate channel intensity statistics	False
Prevent instance overlap	False
Merge regions across diamond model branches	False
Filtering ancestor layer	0
Desired subpixel resolution	
Consider complete pixels	True
Find parent result area	False
Field of view	50
Weight decay	0.0001
Momentum 1	0.9
Momentum 2	0.999
Mini-batch size	20

Parameter	Value
Mini-batches per iteration	20
Iterations without progress threshold	100
Initial learning rate	1
Maximum learning rate in the end of a training run	0.0001
Learning rate shrink factor	0.5
Scale (minimum)	-5
Scale (maximum)	5
Aspect ratio	5
Maximum shear	5
Luminance (minimum)	-5
Luminance (maximum)	5
Contrast (minimum)	-5
Contrast (maximum)	5
Noise	0
JPG compression percentage	0.5
JPG compression quality (minimum)	40
Blur percentage	0.5
Blur maximum pixels	1
Rotation angle (maximum)	180
Rotation angle (minimum)	-180
Flipping	True
A priori weight	0.5
Desired training window size	4
Minimum training window size pixels	32
Maximum training window size pixels	2048
Desired net structure version	Default

Parameter	Value
Width scaler	1
Minimum width	2
Maximum width	4096
